# Ultrafast Patterning Vertically Aligned Carbon Nanotube Forest on Al Foil and Si Substrate Using Chemical Vapor Deposition (CVD)

**DOI:** 10.3390/nano9091332

**Published:** 2019-09-18

**Authors:** Yan-Rui Li, Chin-Ping Huang, Chih-Chung Su, Shuo-Hung Chang

**Affiliations:** 1 Department of Mechanical Engineering, National Taiwan University, Taipei 10617, Taiwan; d00522001@ntu.edu.tw (Y.-R.L.); r92522629@ntu.edu.tw (C.-C.S.); 2 Material and Chemical Research Laboratories, Industrial Technology Research Institute, Hsin-Chu 31040, Taiwan; Chin-PingHuang@itri.org.tw

**Keywords:** carbon nanotubes, patterning, ferrocene, styrene maleic anhydride, ink

## Abstract

This study introduces a method of patterning carbon nanotube (CNTs) forests that is both fast and simple. We found that, as commercially available oil-based markers undergo nanotube synthesis, a thin film forms that prevents the catalyst, ferrocene, from coming into contact with the surface of the test sample. This, thus, blocks CNT growth. Through further deduction, we used styrene maleic anhydride (SMA) to conduct CNT patterning, in addition to analyzing the relationship between the weight percent concentration of the SMA and the extent to which it blocked CNT growth. We developed two separate methods for applying ink to soft and hard substrates: one method involved ink printing and the other laser stripping. In the CNT pattern we produced, a minimum line width of around 10 µm was attained.

## 1. Introduction

Known for their superior electrical, chemical, and mechanical strength, carbon nanotubes (CNTs) possess a vast array of prospective applications. In particular, their abnormally high current-carrying capacity and excellent field emission properties makes them an integral component [1,2,3] in the field of future electronics. The preparation of patterned CNTs also holds development potential for real-life applications, such as field-emission displays [4,5,6,7], CNT sensor chips [8,9,10], probes [11,12,13,14,15,16], and field-effect transistors [17,18,19]. To incorporate CNTs into nanoelectromechanical systems, effective and controllable patterned synthesis of CNTs on specific substrates is crucial. A variety of methods have contributed to the preparation of CNT patterning and are still in use today, including shadow masking, photolithography, electron beam lithography, and soft lithography [20,21,22,23,24,25,26,27,28].

However, all such methods rely on the photolithographic process to produce even the most basic catalyst patterns. Further, such methods rely on the photolithographic process to coat CNTs in unsuitable materials and then synthesize arrays of patterned CNTs. Other methods employ anodic aluminum oxide nano-templates as a growth-limiting template [29] or utilize plasma etching [30,31] for post-synthesis processing. Though not reliant on the photolithographic process, these latter methods are limited to producing arrays that are tubular or fascicular in form and lack the control necessary to create the required patterns. Therefore, there is an urgent need to develop a low-cost CNT patterning process that features high pattern accuracy and quick and simple specimen handling.

This study introduces a method that obviates the need for photolithography and its various time-consuming steps: evaporation, etching, and masking. Instead, it draws on certain polymer materials, transforming them into ink and using the resulting composition to form a barrier between the substrate and the catalyst, ferrocene (Merck, Darmstadt, Germany). This method allows for rapid CNT forest patterning and can be carried out using either ink printing or laser stripping.

## 2. Experiments

This experiment employed a high-temperature furnace to synthesize vertically aligned carbon nanotubes using a three-step chemical vapor deposition (CVD) method. The furnace itself comprised three separate compartments or chambers: the sublimation, transition, and growth chambers; each of which allowed for independent temperature control. All three chambers also contained a heater and a thermocouple, with temperatures set at 250 °C, 400 °C, and 600 °C, respectively. In addition, glass fiber blankets were used for isolation, enabling each chamber to maintain its own distinct temperature. 

Substrates used in the experiment included commercially available household aluminum foil as well as silicon wafers, both of which were cleaned thoroughly with acetone, isopropanol, and deionized water, in that particular sequence, to remove any impurities from the substrate surface. The cleaned aluminum foil was placed on a quartz plate and then loaded into the growth chamber. Next, a quartz boat carrying ferrocene (Merck, Germany) was placed outside the sublimation chamber. Once the reaction began, it was moved into the chamber to undergo the CNT synthesis reaction. When the ferrocene was heated to over 400 °C it pyrolyzed to produce iron and carbon atoms, which were further used in the production of CNT. Specifically, iron atoms were used as catalysts, while carbon atoms constituted a partial carbon source for CNT forests. 

**Figure 1:**
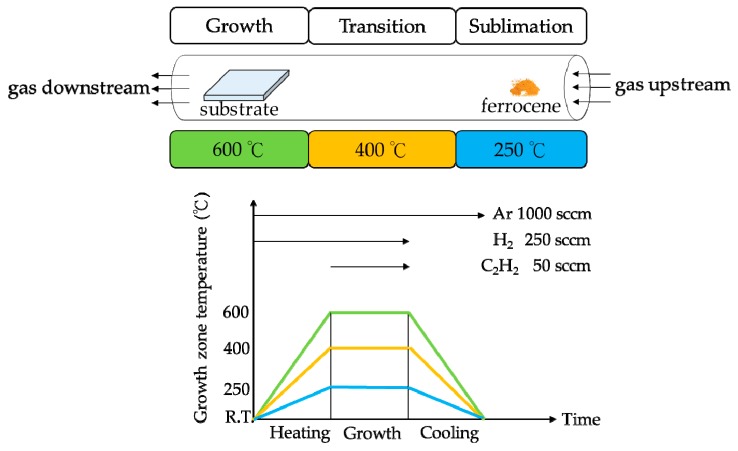
Synthesis setup and recipes for the experiment, which involved a three-step chemical vapor deposition (CVD) process.

During the experiment, as the temperature began to rise, 1000 sccm of Ar and 250 sccm of H_2_ were funneled through a three-inch quartz tube; and the temperature increase duration was set to 20 min to ensure that all three chambers reached their respective temperatures. Next, the tube pumped through 50 sccm of C_2_H_2_, followed by powdered ferrocene, which was pushed through the tube to sublimate, and then moved with the gas flow into the growth chamber for the synthesis reaction, as shown in Figure 1. When the substrate used was aluminum foil, the temperature of the growth chamber was 600 °C; when using silicon wafers, the temperature was 750 °C. No other changes were made. However, the height of the nanotubes were affected by the growth time applied, but this can be adjusted as required.

## 3. Results and Discussions

**Figure 2:**
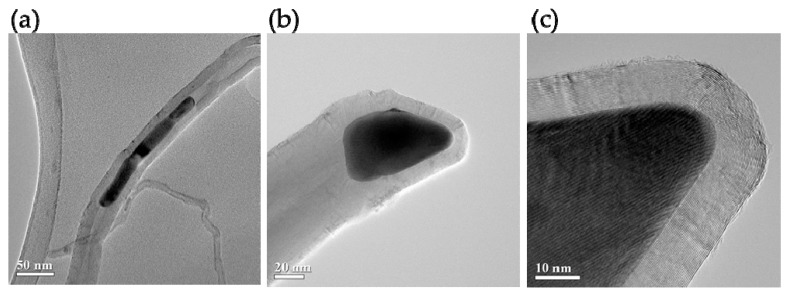
(**a–c**) Transmission electron microscopy images show Fe particles along the length of the carbon nanotube (CNT).

The method adopted in this study was the floating catalyst method [32]. Moreover, the CNTs synthesized were Fe-filled Multi-wall carbon nanotubes. When using aluminum foil as a substrate, the CNTs grew to a height of around 50 µm in 15 min; with silicon as a substrate, they grew to a height of around 250 µm in 15 min. Figure 2 presents a photograph obtained through transmission electron microscopy (TEM; JEOL JEM-2100F, Tokyo, Japan at 200 kV), they have an approximate diameter of tens of nm. 

**Figure 3:**
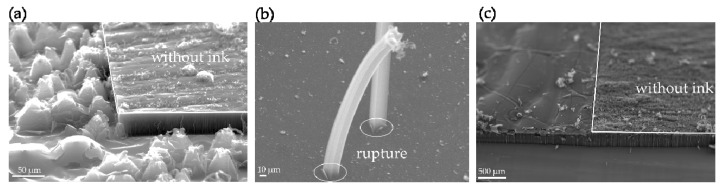
(**a**) A film layer forms on ink-coated areas of the sample following CNT synthesis; the resulting morphology contrasts to the areas without ink. (**b**) Nanotubes grow under the film layer, rupture its surface, and continue growing upward. (**c**) Following synthesis, the area of the sample coated in ink is elevated by a CNT forest growing underneath it.

Through observations made by way of scanning electron microscopy (SEM; JEOL JSM-6390, Tokyo, Japan at 15 kV), we found that the areas of samples coated in ink developed a thin film that directly impacted nanotube morphology, as shown in Figure 3a. Conversely, and as anticipated, CNT forests grew unimpeded in the areas where no ink was applied. We recognized the potential value of this phenomenon and inferred that a specific element that was present in ink caused a thin film to form on the surface of the sample before the ferrocene was sublimated. When the ferrocene reaches the test specimen, the film layer blocks it from coming in contact with the substrate just as the CNT synthesis reaction is set to occur. Moreover, as CNTs display growth selectivity during synthesis, they only grow on certain materials [33]. Therefore, by blocking contact between the catalyst and the substrate surface, the thin film effectively prevents the growth of CNTs.

However, the consistency of the film also determines the degree to which growth is stifled. Thus, we can see that some catalysts still managed to drill through the bottom of the film layer and make contact with the substrate, producing a CNT forest which in turn ruptured the surface of the film. This is clearly evident in Figure 3b, while Figure 3c shows how the entire film structure was elevated by the CNT forest growing below it. We therefore believe that gaining a better grasp on the characteristics of this film layer, to the point of being able to effectively control its behavior, will be of substantial benefit to the process of CNT patterning.

Our initial step was to obtain ink from numerous commercially available, oil-based markers and smear it across aluminum foil to conduct preliminary synthesis testing. (See Figure S1 in Supplementary Materials for details.) In the end, we found that the vast majority of tested inks produced the desired effect. Commercially available inks comprise various elements, including solvents, pigments, resins, and dispersants [34,35]. However, the focus of this study was not to describe the unique formula used by each ink manufacturer and test each composition. Instead, we aimed to verify the efficacy of this new method through a process of deduction and testing. 

**Figure 4:**
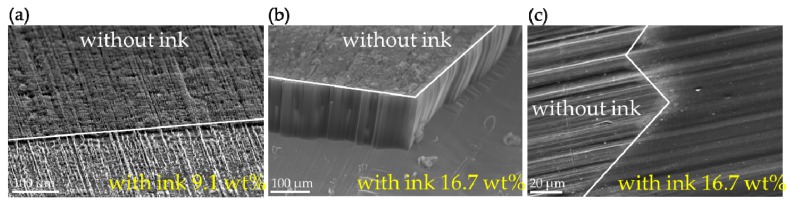
(**a**) At a styrene-maleic anhydride (SMA) concentration of 9.1 wt% nanotubes continue to grow, producing an unclear pattern. (**b**) At a concentration of 16.7 wt%, the pattern is clear and the covering holds. (**c**) Exposed to room temperature conditions, the film layer has already formed prior to CNT synthesis.

To this end, we first consulted a wide sample of related literature and discovered that the combination of styrene and maleic anhydride monomers, as found in the styrene-maleic anhydride (SMA) copolymer, is a commonly-used dispersant found in ink pigments [36,37,38]. We also saw that in research on microcapsules containing phase change materials [39,40], the methanol–melamine–formaldehyde film that formed after mixing with SMA was virtually identical to the film structures we had observed. Thus, we hypothesized that the concentration of SMA would affect the consistency of the film and proceeded to conduct testing and observation using different levels of SMA concentration. As expected, when the SMA was at a lower concentration of 9.1 wt%, as displayed in Figure 4a, its blocking capacity was far more limited, allowing a considerable portion of the CNT forest to push through the surface. This indicates that the ferrocene came in contact with the substrate surface and catalyzed CNT synthesis. Yet when the concentration was increased to 16.7 wt%, as shown in Figure 4b, the thin film produced by the SMA effectively covered the substrate surface, preventing surface ruptures and creating a striking contrast between the ink-covered area and the CNT forest. Through further experiments, we confirmed that the film layer forms prior to CNT synthesis. From Figure 4c we can see that, in room temperature conditions, when the test specimen has been covered with a coat of SMA ink, the thin layer of film has already formed and produced visible marks from clinging to the substrate and sample surfaces.

**Figure 5:**
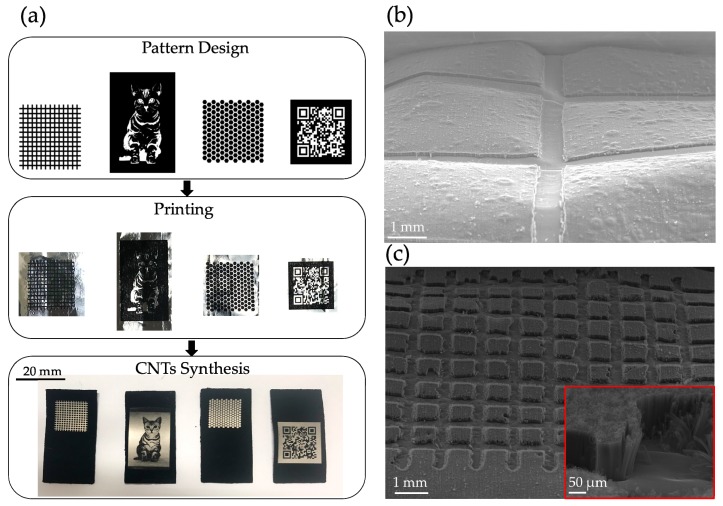
(**a**) Ink printing was used to pattern CNT forests on aluminium foil (**b**) and the horizontal and vertical stripe patterns from Figure 5**a** were observed via scanning electron microscopy (SEM) images. (**c**) Reducing line width caused ink splattering and diminished the ability of ink to block CNT forest growth.

After better understanding the characteristics of the film layer, we devised two separate methods that could be applied respectively to the soft substrate of aluminum foil and the hard substrate of silicon wafer. With the soft substrate, we injected the ink composition into a regular household printer with a continuous ink supply system (CISS). We then tightly pressed aluminum foil to a piece of A4 paper so that it fed smoothly into the printer without causing wrinkles or paper jams. After designing the required patterns using computer software, we simply printed them onto aluminum foil and loaded them into the chemical vapor deposition (CVD) system to synthesize. The process is outlined in Figure 5a. For more details, please refer to Figure S2 in the Supplementary Materials. 

As can be seen from the above description, the process is both quick and convenient. From our observation of horizontal and vertical stripes, shown in Figure 5b, we can see that in areas where the ink was applied, CNT growth was effectively blocked. Yet our attempts to reduce the line width were unsuccessful, as the ink used was different to the ink that originally came with the printer. The result is shown in Figure 5c. Severe ink splattering affected the resolution, and the printer was unable to keep the ink within the outline of the initial patterns. Thus, at present, this method is applicable to millimeter-sized patterns and substrates that are flexible and thin.

Substrates like silicon wafers lack pliability and cannot be fed into commercially sold printers to undergo ink printing. Yet silicon is one of the main materials used in the semiconductor industry right now. To solve this dilemma and enhance pattern accuracy, we developed an alternate approach that proved to be even more effective and led to vastly enhanced pattern accuracy. 

**Figure 6:**
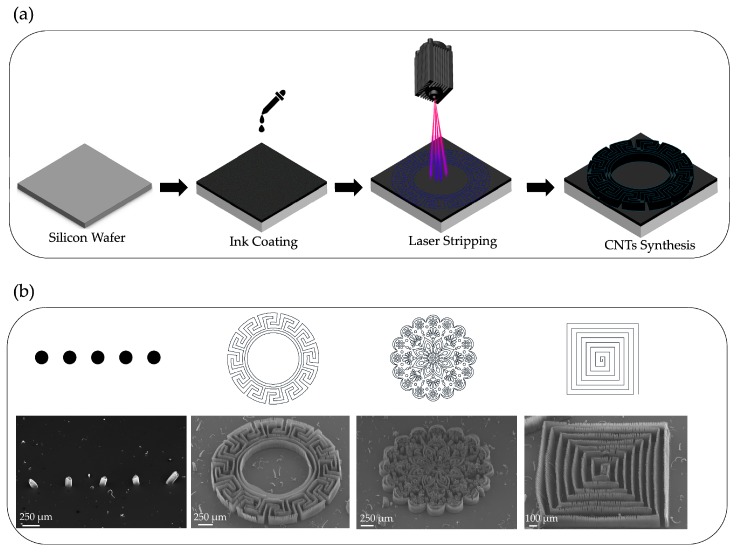
(**a**) The process of patterning CNT forests on a silicon wafer using laser stripping. (**b**) SEM was used to observe a synthesized CNT forest pattern with a minimum line width of 10 µm.

This method utilizes laser stripping. The silicon is coated in ink and a UV laser (Taiwan 3 Axle Technology-TAUV using Nd:YVO_4_ 355 nm, Taichung, Taiwan) is applied, not for image projection or contact masking, but to create a pattern by using the direct writing system to remove the ink. This is followed by CNT synthesis, which causes nanotube growth in the areas removed by the laser and renders a patterned effect. The process is outlined in Figure 6a and described in more detail in Figure S3 of the Supplementary Materials. With laser stripping, pattern accuracy is determined by the spot size of the laser. Moreover, specimen preparation is simple and fast, and a line width of just 10 µm can be achieved. Figure 6b shows SEM images of patterns we designed with this approach. As can be clearly seen, the resulting nanotube patterns were extremely successful.

## 4. Conclusions

In summary, we found that commercially available oil-based ink markers contain a polymer helpful for patterning CNTs. This polymer is known as SMA and is a common dispersant in ink. We used SMA in testing and came up with two methods for more effectively patterning CNTs: ink printing and laser stripping. The results of subsequent experiments were successful. Using the laser stripping method, we were able to produce intricate CNT patterns with line widths of just 10 µm. Aside from being extremely cost-effective, both methods require minimal preparation, are quick and easy to carry out, and obviate the countless complex steps associated with conventional methods of photolithography. Our approach offers ample opportunities for large-scale commercialization.

This study proposed new methods of CNT patterning that hold vast potential. Further, our conclusions verified the validity of these methods by way of experimentation. To conclude, we hereby outline some relevant items for consideration and posit possible directions for future research:

1. The initial ink printing method turned up several issues, including how to smoothly feed aluminum foil into the printer, how to avoid ink splattering, and how to enhance pattern resolution. If equipment was developed that addressed these issues, the ink printing method would have a much broader application.
2. If using a laser with a smaller spot size, the line width of the patterns could be reduced even further, resulting in an increase to the concentration of nanotube forest arrays and an improvement in application.
3. The laser stripping method is equally applicable to thin and highly pliable materials, such as aluminum foil, as long as the parameters are adjusted properly so that the aluminum surface is not damaged when the ink is removed.
4. Apart from SMA, we believe that there are other materials that could equally achieve the task of blocking CNT forest growth. Any polymer that does not carbonize under high heat and possesses high melt viscosity could be employed to achieve CNT patterning. As nanotube synthesis involves immense heat, materials with low viscosity and high fluidity could affect the overall integrity of the pattern.

## Supplementary Materials

The following are available online at https://www.mdpi.com/2079-4991/9/9/1332/s1, Figure S1: Oil-based markers to conduct preliminary synthesis testing, Figure S2: The specimen preparation process for CNT patterning using inkjet printers, Figure S3: Laser stripping to remove the ink from the surface of the sample
