# Supplementary material for: Ultrafast Patterning Vertically Aligned Carbon Nanotube Forest on Al Foil and Si Substrate Using Chemical Vapor Deposition (CVD)
Source: Nanomaterials (Basel). 2019 Sep 18;9(9):1332. doi: 10.3390/nano9091332 (PMC6781085; doi:10.3390/nano9091332)
Supplement: Supplementary file 1 [file nanomaterials-09-01332-s001.pdf]

nanomaterials

Article

Ultrafast Oatterning Vertically Aligned Carbon Nanotube Forest on Al Foil and Si Substrate Using Chemical Vapor Deposition (CVD)

Yan-Rui Li [1], Chin-Ping Huang [2], Chih-Chung Su [1] and Shuo-Hung Chang [1,*]

[1] Department of Mechanical Engineering, National Taiwan University, Taipei 10617, Taiwan; d00522001@ntu.edu.tw (Y.-R.L.); r92522629@ntu.edu.tw (C.-C.S.)
[2] Material and Chemical Research Laboratories, Industrial Technology Research Institute, Hsin-Chu 31040, Taiwan; Chin-PingHuang@itri.org.tw (C.-P.H.)
* Correspondence: shchang@ntu.edu.tw (S.-H.C.); Tel.: +886-2-3366-9421

Received: 13 August 2019; Accepted: 16 September 2019; Published: date

Supplementary Figures S1-S3

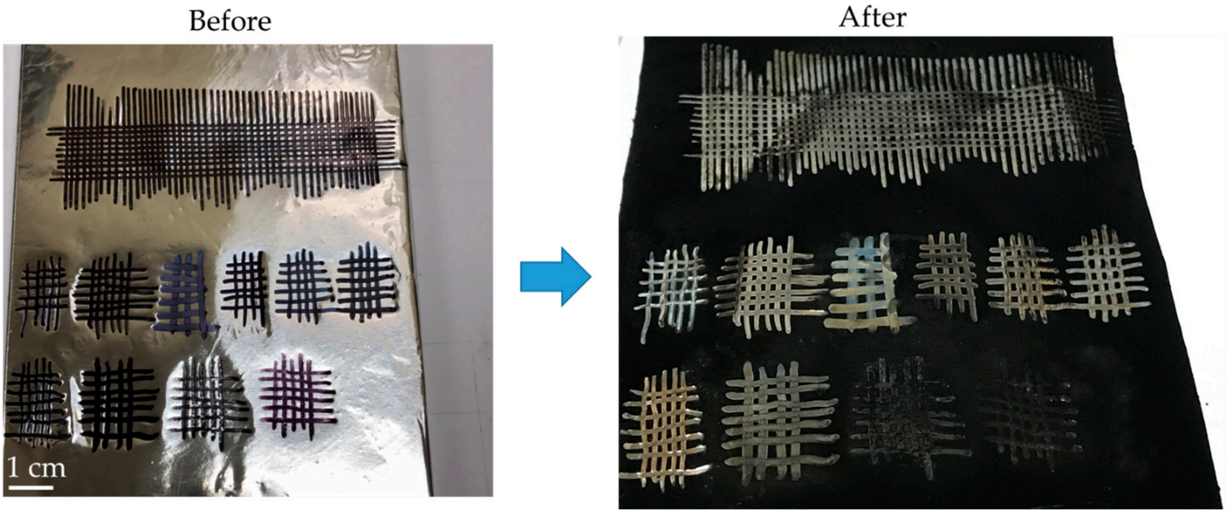

Figure S1. The left image shows the samples prior to nanotube growth. We used inks from a variety of manufacturers and made sure that every pattern was coated in a separate type of ink. The right image displays the post-growth samples; we can see that each one has a disparate color and that some of the inks were unable to block CNT growth.

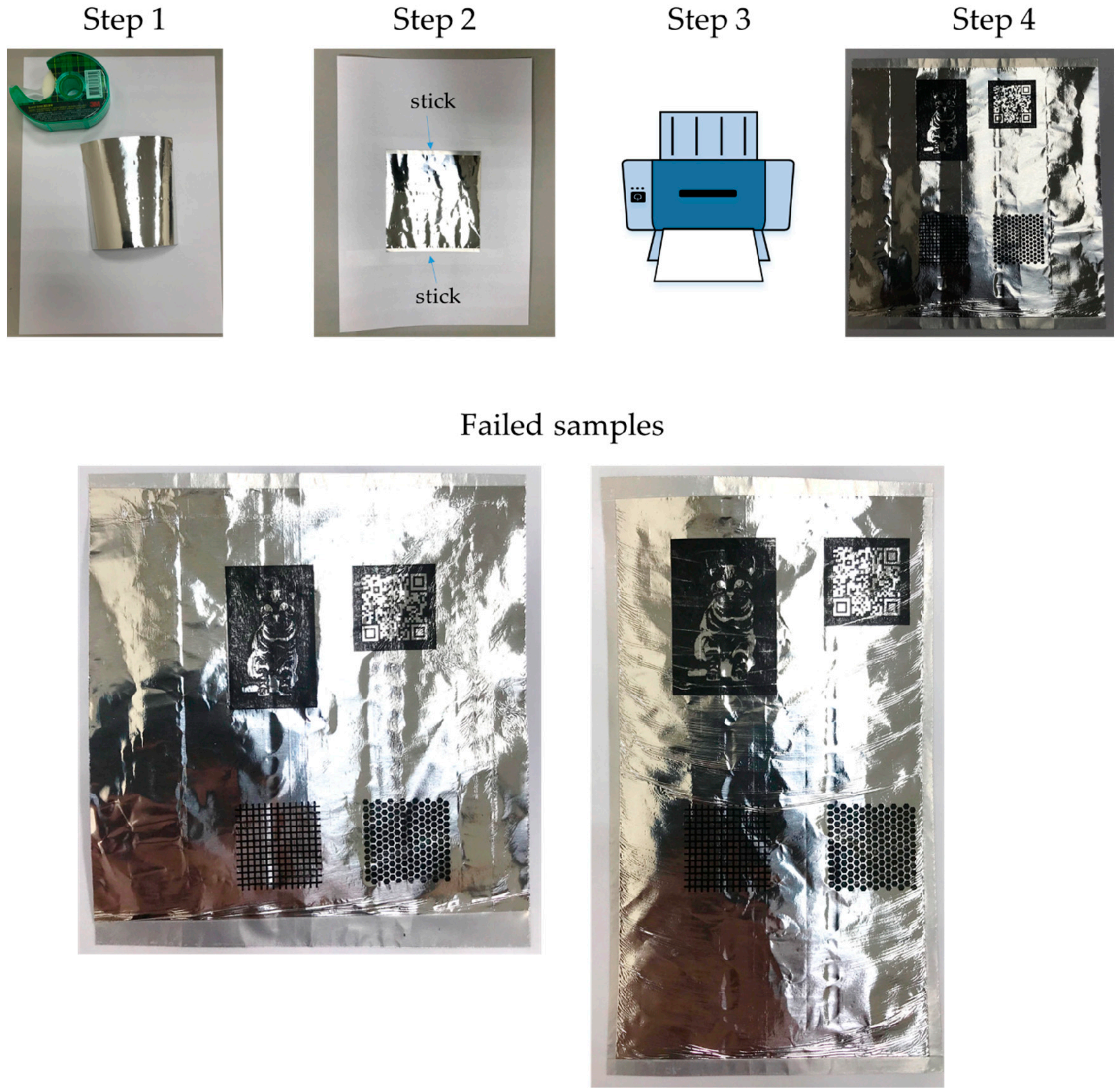

Figure 2. The specimen preparation process for CNT patterning using inkjet printers.

Step 1: Prepare a piece of A4 paper, several strips of wall-safe tape, and a roll of kitchen aluminum foil.

Step 2: Using wall-safe tape, carefully affix aluminum foil to the piece of A4 paper, making sure to apply tape only to the top and bottom of the page, not the sides, so as to avoid paper jams when inserting into the printer. The aluminum foil must be clean, otherwise the ink will not stick; so, avoid all contact with hands.

Step 3: Regular inkjet printers are unsuited to this method, as they contain sealed ink cartridges into which our SMA ink composition cannot be injected. Thus, if using a traditional inkjet printer, please first have the printer modified so that it is equipped with CISS. Presently, many companies provide printer modification services.

Step 4: After using a computer to design the pattern, simply print the pattern onto the aluminum foil.

Be aware of the position arrangement of the patterns. From the 'Step 4' image (above) we can see the creases that develop on aluminum foil when squashed by the paper input system. Please also

refer to the failed samples above. Try to avoid placing the patterns along these crease lines. Additionally, keep in mind that the print head nozzle is unable to print such patterns in large volumes. As the SMA ink we personally used was not the original ink used in the printer and was just a resin component, after several uses the print head nozzle malfunctioned and had to be replaced. Thus, due to equipment limitations, the method is currently uneconomical and requires a machine specifically built for this purpose.

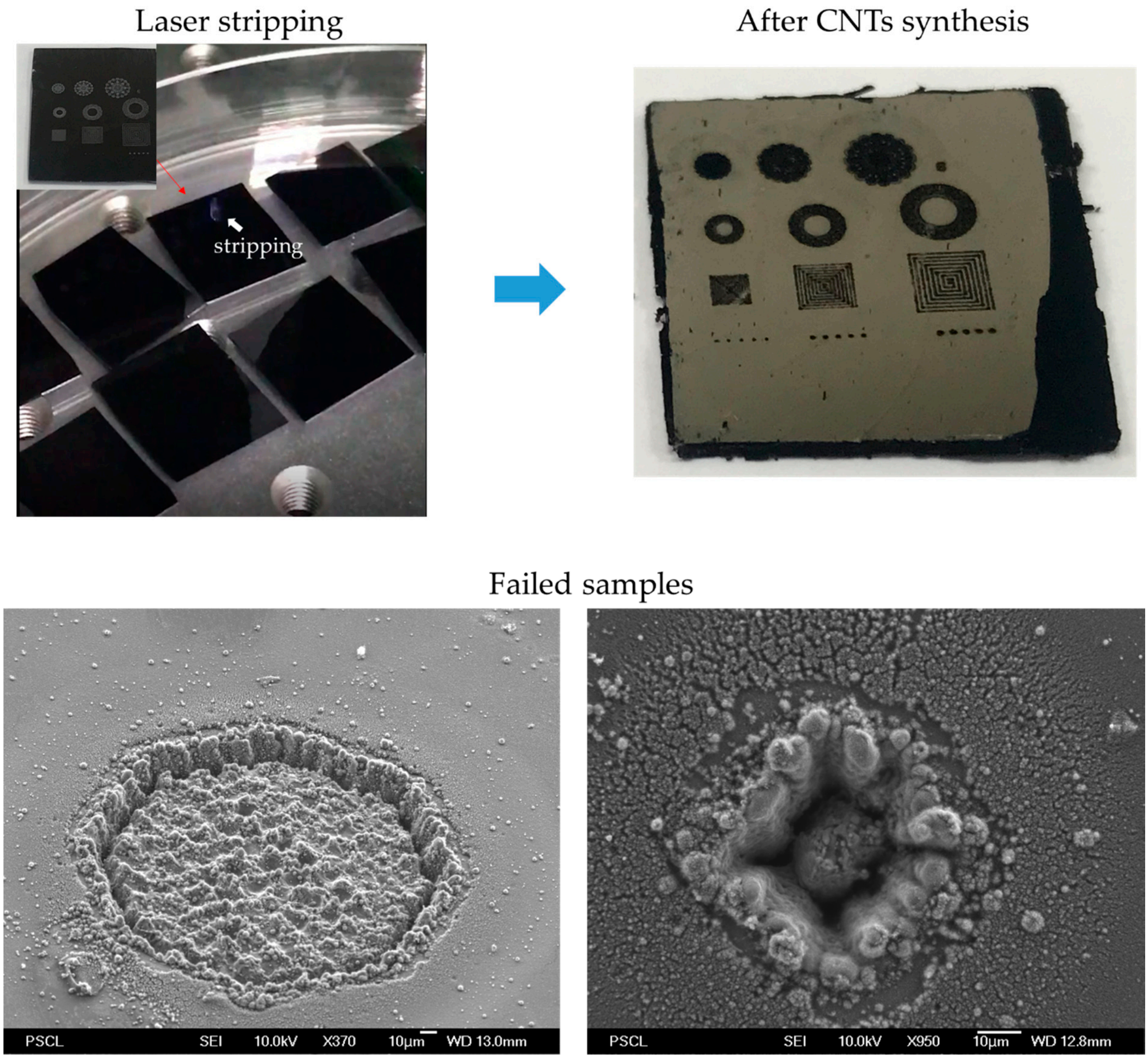

Figure 3. After applying laser stripping to remove the ink from the surface of the sample, CNT synthesis is carried out to complete the patterning. During this process, pay particular attention to the parameter adjustments of the laser. If the laser energy absorbed by the sample is too strong, it will create cavities on the silicon surface, as seen in the failed samples above, and result in substandard patterns upon completion of CNT synthesis.

© 2019 by the authors. Submitted for possible open access publication under the terms and conditions of the Creative Commons Attribution (CC BY) license (http://creativecommons.org/licenses/by/4.0/).
